# Screening of the highly pathogenic *Beauveria bassiana* BEdy1 against *Sogatella furcifera* and exploration of its infection mode

**DOI:** 10.3389/fmicb.2024.1469255

**Published:** 2024-11-25

**Authors:** Xing Xiang, Siyuan Yu, Andrews Danso Ofori, Shuhua Liu, Qunfang Yang, Jing Shang

**Affiliations:** ^1^College of Agronomy, Sichuan Agricultural University, Chengdu, China; ^2^Agricultural and Rural Bureau, Jiajiang County, Leshan, China

**Keywords:** *Beauveria bassiana*, WBPH, infection patterns, biological control, pathogenicity

## Abstract

The white-backed planthopper (WBPH, *Sogatella furcifera*) is a notorious pest affecting rice production in many Asian countries. *Beauveria bassiana*, as the most extensively studied and applied insect pathogenic fungus, is a type of green and safe biological control fungus compared to chemical insecticides, and it does not pose the “3R” problem. In this study, the strain BEdy1, which had better pathogenicity to WBPH, was screened out from eight strains of *B. bassiana*. The daily growth rate, sporulation, and germination rate of BEdy1 strain were 3.74 mm/d, 1.37 × 10^8^ spores/cm^2^, and 96.00%, respectively, which were significantly better than those of other strains. At a concentration of 1 × 10^8^ spores/mL, the BEdy1 strain exhibited the smallest LT_50_ value (5.12 d) against the WBPH, and it caused the highest cumulative mortality and muscardine cadaver rates of the pest, which were 77.67 and 57.78%, respectively. Additionally, BEdy1 exhibited a significant time-dose effect on WBPH. This study further investigated the pathogenic process of BEdy1. The results showed that BEdy1 invaded by penetrating the body wall of the WBPH, with its spores mostly distributed in the insect’s abdominal gland pores, compound eyes on the head, and other locations. At 36 h, the germinated hyphae penetrated the insect’s body wall and entered the body cavity. At 84 h, the hyphae emerged from the body wall and accumulated in the insect’s abdomen, leading to a significant number of insect deaths at this stage. At 120 h, the hyphae entangled the insect’s compound eyes and produced new conidia on the insect’s body wall, entering a new cycle of infection. These findings indicate that BEdy1 has a strong infection ability against WBPH. In summary, this study provides a new highly pathogenic strain of *B. bassiana*, BEdy1, for the biological control of WBPH, which is of great significance for the green prevention and control of rice pests.

## 1 Introduction

The white-backed planthopper [*Sogatella furcifera* (Horváth) (Hemiptera, Delphacidae)] is a significant rice pest, primarily feeding on rice plants by clustering as adults and nymphs at the base of the rice stems (Jin et al., 2021). The WBPH directly pierces and sucks the phloem sap of rice plants, and excessive feeding can lead to slowed plant growth, reduced seed setting rates, and even plant death, severely impacting rice yields (Zhu et al., 2019). Additionally, the WBPH can transmit the Southern rice black-streaked dwarf virus (SRBSDV), causing secondary damage to rice plants (Jia et al., 2021). Studies have also shown that the honeydew excreted by the WBPH is rich in various amino acids, which promotes the growth of the rice sheath blight pathogen, resulting in combined damage from the insect and the disease, thereby exacerbating losses in agricultural production (Zhang Y. X. et al., 2014).

Currently, the prevention and control of WBPH in production still primarily relies on chemical methods, such as using imidacloprid, buprofezin, thiamethoxam, dinotefuran, and sulfoxaflor (Mu et al., 2016; Ali et al., 2019; Li et al., 2021; Kang et al., 2022). However, the long-term and extensive unreasonable use of chemical pesticides not only causes environmental pollution but also harms natural enemies, while also making WBPH more prone to developing resistance (Zhang K. et al., 2014). On the other hand, microbial pesticides are environmentally friendly and do not pose “3R” issues (resistance, residue, and resurgence) (Peng et al., 2020). Therefore, to achieve pesticide reduction and efficacy enhancement in rice production, it is of great significance to develop safe and efficient biological pesticides (living organisms and their produced active substances).

*B. bassiana* (Hypocreales: Clavicipitaceae) is a harmless fungus widely present in nature (Li et al., 2012). As one of the currently widely used live microbial pesticides, *B. bassiana* has a broad host range and can parasitize over 700 species of insects. For instance, *B. bassiana* is effective in controlling pests such as *Polyphylla fullo* (Linnaeus), *Monochamus alternatus*, *Frankliniella occidentalis*, and *Lissorhoptrus oryzophilus* (Erler and Ates, 2015; Guo et al., 2020; Kim et al., 2014; Skinner et al., 2012). In addition, *B. bassiana* is also commonly used to control the brown planthopper (Li et al., 2014; Song and Feng, 2011). Lee et al. (2015) found that after spraying *B. bassiana* ERF836 at a concentration of 1 × 10^7^ conidia/mL for 7 days, the cumulative mortality rate of the brown planthopper reached 85%. Feng and Pu (2005) reported that treating nymphs of the brown planthopper with *B. bassiana* SG8702 at concentrations ranging from 1 × 10^6^ to 1 × 10^8^ spores/mL resulted in mortality rates of 32 to 91%. *B. bassiana* has been commercially developed as an environmentally friendly biological pesticide (Zimmermann, 2007). Therefore, this study aims to screen for effective strains of *B. bassiana* against the WBPH, in order to provide new materials for the green prevention and control of the WBPH.

In recent years, some researchers have explored the infection patterns and histopathology of *B. bassiana* on insects (Ferreira et al., 2024; Marzouk and Ali, 2023). When *B. bassiana* infects insects, its conidia germinate to produce germ tubes, which subsequently form structures such as appressoria and penetration pegs (Duan et al., 2017). *B. bassiana* invades the interior of the insect host through the penetration of its cuticle by germ tubes and appressoria, absorbing nutrients from the hemocoel for growth and reproduction. The mechanical pressure and toxins generated by the hyphae damage the host’s internal organs, ultimately leading to insect death (Caloni et al., 2020). A study by Ibrahim et al. (2019) found that within 3–4 days of *B. bassiana* infection, its conidial bundles, under mechanical pressure, directly penetrate the intersegmental cuticle of pear psyllas, followed by hyphal invasion of all internal organs until the insect’s death (Ibrahim et al., 2019). Shen et al. (2018) through scanning electron microscopy and transmission electron microscopy observations, discovered that the conidia of *B. bassiana* directly enter the body of *Antheraea pernyi* pupae from its wall, subsequently eroding its fat cells until the pupa hardens and dies. Additionally, *B. bassiana* can secrete extracellular degradative enzymes during infection, which include proteases, chitinases, and esterases (Fang et al., 2009). Reports indicate that chitinases not only function as hydrolytic enzymes to destroy the insect’s cuticle but also act as inducible enzymes expressed within the host (Li et al., 2014). However, research on the infection patterns and histopathological changes of *B. bassiana* in WBPH has not yet been reported.

Our research group has previously conducted studies on the growth and development of WBPH under sublethal concentrations of *B. bassiana* strain BEdy1, which was isolated from the *Ergania doriae yunnanus* (Xia et al., 2023). This paper intends to further compare the basic biological characteristics of the eight collected strains of *B. bassiana* (including the BEdy1 strain) and evaluate their pathogenicity to WBPH. Simultaneously, the infection process of the BEdy1 strain on the surface and inside the body of WBPH will be observed through microscopic techniques. This experiment will provide new insights into the biological control of WBPH, which is of great significance for the green prevention and control of rice pests.

## 2 Materials and methods

### 2.1 Insects and strains tested

*S. furcifera* was collected from a rice field in Hongyanba (105.4756° E, 28.1880° N), Xuyong County, Luzhou City, Sichuan Province, China, in 2020. It was then reared on rice (TN1) seedlings (approximately 15 cm tall) in the intelligent artificial climate chamber (RXE-450, Ningbo Jiangnan Instrument Factory Co., Ltd., Ningbo, China) for over 15 generations without exposure to any pesticides. The chamber was maintained at 26 ± 1°C, with a relative humidity of 70 ± 10%, and a photoperiod of 14 h: 10 h (L:D).

Test strains (Specific information is provided in Table 1). The *B. bassiana* strain BEdy1 used in the test was provided by Shang Jing’s research group from the Phytopathology Laboratory, College of Agronomy, Sichuan Agricultural University. BEdy1 (GenBank: MK345993) was isolated from the body of the soybean pest, *Ergania Doriae Yunnanus*, collected from a soybean field in Renshou County, Sichuan Province, in September 2018. A BLAST comparison of the 544 bp rDNA-ITS of the strain revealed that BEdy1 shared 99% similarity with strain JEF006 (GenBank: KT280276). Combining these results with morphological observations of the strain, the pathogen causing field mortality of *Ergania Doriae Yunnanus* was ultimately identified as *B. bassiana* BEdy1 (Zhang et al., 2020). The *B. bassiana* JZ21004 is a commercial strain from Hubei QIMING BIO Engineering Co., Ltd. The remaining six *B. bassiana* were isolated and identified from muscardine cadavers collected in the field. These test strains were cultured on PPDA medium (consisting of 200 g potato, 20 g dextrose, 20 g agar, and 10 g peptone) in plastic Petri dishes (90 mm diameter), which were then wrapped with Parafilm and placed in darkness at 26 ± 1°C. Each strain was inoculated onto slant culture medium and stored at 4°C for future use, with additional preservation in 50% glycerol at −80°C in an ultra-low temperature freezer.

**Table 1:** Fungal strains used in the test.

Strain	Geographic origin	Host insects	Accession number (NCBI)
BEdy1	Renshou, Sichuan	*Erganiadoriae yunnanus*	MK345993
WJGP1	Wenjiang, Sichuan	*Cyrtorhinus lividipennis* (Reuter)	OM902633
WJGP3	Wenjiang, Sichuan	*Coccinella septempunctata*	OM902631
WJGP5	Wenjiang, Sichuan	*Myzus persicae* (Sulzer)	OM902632
CZ9	Chongzhou, Sichuan	*Musca domestica*	OM902630
WJGP11	Wenjiang, Sichuan	*Cryptotympana atrata* (Fabricius)	OM902629
WJGP15	Wenjiang, Sichuan	*Nephotettix cincticeps* (Uhler)	OM902634
JZ21004	QIMING BIO Engineering co., Ltd	Strain used in production	Unknown

### 2.2 Comparison of growth, spore production, and spore germination of different *B. bassiana* strains

Comparison of colony growth rates (Li et al., 2014). A 5 mm diameter fungal plug was taken from the reserved *B. bassiana* culture medium and placed at the center of a new culture medium. The cultures were then incubated in a dark incubator at 26°C with three replicates set up for each strain. On the 3rd and 7th days, the diameters of the colonies were measured in both vertical and horizontal directions, and their average values were taken to calculate the daily average growth rate of the colonies (mm/d).

Comparison of initial sporulation time and sporulation yield (Li et al., 2014). After the mycelium grew on the culture medium, a small amount of mycelium was collected daily using a colorless, transparent adhesive tape. Under the microscope, we observed the presence of conidia and recorded the initial sporulation time. After 15 days of cultivation, six 6 mm diameter fungal plugs were taken from the midpoint between the center and the edge of the colony in different directions and placed into 10 mL centrifuge tubes containing 5 mL of 0.05% Tween-80 sterile aqueous solution. The tubes were then shaken on a thermostatic oscillator for 30 min to obtain a spore suspension. Subsequently, the spore content in the spore suspension was observed under a microscope using a hemocytometer and converted to the number of spores per cm^2^ of colony. Each strain was tested in triplicate, and the average values were taken.

Comparison of spore germination rates (Li et al., 2014). After thoroughly washing the conidia on the PPDA plate with 0.05% Tween-80 solution, a spore suspension of 1.0 × 10^7^ spores/mL was prepared and incubated in a constant temperature oscillator at 25°C and 150 rpm for 24 h. The spore germination of each strain (spore tube larger than the short radius of the spore is considered germination) was observed using a blood cell counting plate, and the spore germination rate was calculated by examining five different fields of view under a microscope. Each strain was set to four replicates and the average value was calculated.

### 2.3 Pathogenicity testing of different strains of *B. bassiana* against WBPH nymphs

The spray method was used to screen highly virulent strains against 3rd instar nymphs of WBPH (Lin et al., 2013). All eight strains were prepared into spore suspensions at a concentration of 1 × 10^8^ spores/mL. Rice seedlings at the 4–5 leaf stage were washed with clean water, dried, and then grouped into sets of 20 plants. Each set was inoculated with 30 third-instar nymphs in the mid-stage. A handheld sprayer was used to uniformly apply 3 mL of the spore suspension. A 0.05% Tween-80 solution served as the control. The seedlings were then placed in an artificial climate chamber (*T* = 27°C ± 1°C, RH = 75% ± 5%, L: *D* = 14: 10 h) for rearing, and the rice seedlings were replaced every 3 days. The number of dead WBPH in each treatment was counted daily within 10 days (gently touching the insect body with a brush, using the criteria of weakness and inability to crawl normally as the standard for death). The dead insect bodies were maintained under moist conditions, and fungi were isolated and identified from their body surface and hemocoel, respectively. Finally, the number of mummified insects was recorded. Both the treatments and the control were set up in triplicate.

Retesting of the screened strains with the highest pathogenicity. The concentration gradient of spore suspension is set as follows: 1.0 × 10^5^ spores/mL, 1.0 × 10^6^ spores/mL, 1.0 × 10^7^ spores/mL, 1.0 × 10^8^ spores/mL, and 1.0 × 10^9^ spores/mL. Using 0.05% Tween-80 solution as a control, the number of deaths from WBPH was counted daily within 10 days of spraying. The treatment and control groups were set to three replicates.

### 2.4 Fluorescence microscopy observation of WBPH infection by *B. bassiana* BEdy1

Fluorescence staining was used to observe the invasion process of *B. bassiana* in the nymphs of WBPH (Wang et al., 2015). 40 mg of fluorescein dye FDA (Solarbio Company) was dissolved in 10 mL of acetone to form an FDA stock solution. 88 μL of the FDA stock solution was then diluted in 10 mL of deionized water to prepare the FDA working solution, which was stored in a dark, cold place for later use. The inoculation method was the same as described in section 2.3. Samples were collected at 12 h, 24 h, 36 h, 60 h, 84 h, and 108 h post-inoculation, with one test insect selected at each time point. Before observation, the samples were placed on clean slides and treated with the FDA working solution, allowing them to react in the dark for 5 min. Subsequently, the infection status of nymphs by *B. bassiana* BEdy1 at each time point was observed using a fluorescence microscope with a GFP filter, and photographs were taken for recording.

### 2.5 Scanning electron microscopy observation of WBPH infection by *B. bassiana* BEdy1

The method of Toledo et al. (2010) was adopted with slight modifications. The inoculation procedure was the same as described in section 2.3. Samples were collected at 12 h, 24 h, 72 h, 96 h, and 120 h post-inoculation, with one test insect selected at each time point. Sterile water with 0.05% Tween-80 served as the control. After sampling, the samples were fixed in 2.5% glutaraldehyde at 4°C for 24 h and then washed three times with 0.1 mol/L phosphate buffer for 10 min each time to ensure complete removal of the fixative. Subsequently, the samples were fixed in 1% osmic acid for 2 h and then dehydrated in a graded series of 30, 50, 70, 90, and 100% ethanol for 15 min each. After dehydration, the samples were air-dried at room temperature for 2 h. They were then mounted on a scanning electron microscope (SEM) stub using conductive adhesive tape, vacuumed, and coated with gold. Observations were made under the SEM at appropriate positions and magnifications, and photographs were taken for recording.

### 2.6 Histopathological observation of WBPH infection by *B. bassiana* BEdy1

The method of Toledo et al. (2010) was adopted with slight modifications. The inoculation procedure was the same as described in section 2.3. Samples were collected at 60 h, 72 h, and 96 h post-inoculation, with one test insect selected at each time point. Sterile water with 0.05% Tween-80 served as the control. The samples were fixed in Bouins fixative at 4°C for 24 h, followed by graded dehydration in 35, 50, 70, 80, 90, 95, and 100% ethanol for 1 h each. Subsequently, the samples were infiltrated in 50, 70, 85, and 100% xylene-ethanol solutions and 50% paraffin-xylene for 1 h each, and then immersed in paraffin at 60°C for 2 h. After tissue embedding, sections of the thorax and abdomen of the insect were cut using a rotary microtome with a thickness of 8 μm. Following spreading in a 35°C water bath, the sections were picked up using adhesive slides, dried in a 37°C oven, and stained according to the instructions of the Hematoxylin and Eosin (HE) staining kit. They were then dehydrated in a series of ethanol concentrations, mounted with neutral balsam, and dried at 37°C. Subsequently, histopathological changes in the WBPH infected by *B. bassiana* were observed under an optical microscope, and photographs were taken for recording.

### 2.7 Data analyses

The average growth rate, initial spore production time, spore yield, spore germination rate, cumulative corrected mortality, and muscardine cadaver rates of the strains were analyzed for significant differences using SPSS 22.0. Duncan’s new multiple range method was used for multiple comparisons of the means. Using the Probit method of POLO 2.0 software to calculate the LT_50_ and LC_50_. The line chart was drawn using Graphpad Prism 9.5 software. The formula used for pathogenicity testing is as follows:

$$\begin{array}{l} \mathrm{Mortality}\left(\%\right)=\\ \frac{\mathrm{Number of live insects before treatment}-\mathrm{Number of live insects after treatment}}{\mathrm{Number of live insects before treatment}}\times 100 \end{array}$$

$$\begin{array}{l} \mathrm{Corrected mortality}\left(\%\right)=\\ \frac{\mathrm{Mortality of the treatment group}-\mathrm{Mortality of the control group}}{1-\mathrm{Mortality of the control group}}\times 100 \end{array}$$

$$\mathrm{Muscardine cadaver rate}\;\left(\%\right)=\frac{\mathrm{Number of muscardine cadavers}}{\mathrm{Number of tested insects}}\times 100$$

## 3 Results

### 3.1 Comparison of colonial characteristics among different strains

Through observation, it was found that the surface of most colonies appeared white or milky white, while a few were powdery white or yellowish-white; the reverse side was mostly light yellow (Table 2). Additionally, some colonies exhibited concentric patterns or groove-like lines. Based on the appearance of different *B. bassiana* on PPDA medium, they can be roughly classified into three types: powdery, flocculent, and tomentose colonies (Table 2). The strains BEdy1, JZ21004, and WJGP15 are spore-forming strains with thin, powdery colonies and high spore production (Table 2). The mycelial strains (WJGP3, CZ9) have flocculent colony morphology and produce fewer spores (Table 2). The colonies of WJGP1, WJGP5, and WJGP11 are tomentose, with white or milky white spore powder. Among them, WJGP11 has radial folds and aerial hyphae appear in the later stages (Table 2).

**Table 2:** Colony characteristics of the strains of *B. bassiana*.

Isolate	Colony morphology	Color of colony	Color of substrate	Color of spore
BEdy1	Thin powdery	White	Light yellow	Milky white
WJGP1	Woolly	White	Pale yellowish white	Milky white
WJGP3	Flocculent	Powdery white	Powdery yellow	White
WJGP5	Woolly	Milky white	Light yellow	White
CZ9	Flocculent	White	Light yellow	White
WJGP11	Woolly	White	Light yellow	White
WJGP15	Thin powdery	Yellowish	Yellowish brown	Milky white
JZ21004	Thin powdery	Yellowish	Light yellow	Milky white

### 3.2 Comparison of growth, spore production, and spore germination among different strains

The vegetative growth rate of strains is one of the indicators reflecting their excellent characteristics. Vegetative growth measurements were conducted for eight strains, and the results showed significant differences in colony growth rates among the different test strains (Table 3). Among them, the BEdy1, WJGP1, and WJGP11 strains grew faster, with a daily average increase in colony diameter greater than 3.3 mm, which was significantly higher than that of the WJGP3, WJGP5, CZ9, and WJGP15 strains (*p* < 0.05).

**Table 3:** The biological characteristics of eight different strains of *B. bassiana.*

Strains	Average daily colony diameter increase (mm/d)	Time of producing spores (d)	Sporulation (×10^7^ spores /cm^2^)	Germination rate (%)
BEdy1	3.74 ± 0.24^a^	5.67 ± 0.58^bc^	13.70 ± 0.40^a^	96.00 ± 0.45^a^
WJGP1	3.50 ± 0.06^ab^	4.67 ± 0.58^c^	7.09 ± 0.98^c^	91.45 ± 0.64^c^
WJGP3	2.59 ± 0.15^cd^	7.00 ± 1.00^ab^	3.06 ± 1.13^d^	82.30 ± 0.65^f^
WJGP5	2.55 ± 0.59^cd^	7.33 ± 1.15^ab^	6.79 ± 0.66^c^	91.65 ± 0.66^c^
CZ9	1.93 ± 0.12^e^	7.67 ± 0.58^a^	1.48 ± 0.15^e^	85.93 ± 0.84^e^
WJGP11	3.34 ± 0.17^ab^	7.00 ± 0.00^ab^	5.76 ± 0.32^c^	87.73 ± 0.50^d^
WJGP15	2.11 ± 0.23^de^	6.33 ± 1.15^ab^	8.64 ± 1.03^b^	94.15 ± 0.85^b^
JZ21004	3.02 ± 0.23^bc^	5.67 ± 1.15^bc^	9.90 ± 0.75^b^	83.15 ± 1.14^f^

Data are presented as mean ± SE; values with different lowercase letters indicate significant differences at 0.05. The same below.

The time required for the WJGP1 strain to start sporulation (4.67 days) was significantly shorter than that of the WJGP3, WJGP5, CZ9, WJGP11, and WJGP15 strains (*p* < 0.05), and was 3 days earlier than the latest sporulation producing CZ9 strain (7.67 days), but there was no significant difference compared to the BEdy1 and JZ21004 strains (*p* > 0.05). In addition, there were significant differences in spore production among different strains, with BEdy1, JZ21004, and WJGP15 strains producing significantly more spores than the other tested strains (*p* < 0.05). Among them, BEdy1 strain had the highest spore production, reaching 1.37 × 10^8^ spores/cm^2^, which was 9.26 times the spore production of loose hyphal CZ9 strain (1.48 × 10^7^ spores/cm^2^).

The germination rate of spores is also an indicator of the vitality of strains. The results showed that there were significant differences in the germination rates of spores among the different strains (Table 3). The BEdy1 strain had a germination rate of 96% at 24 h, which was significantly higher than that of other strains (*p* < 0.05). Based on the comprehensively measured indicators, the spore production and spore germination rate of the BEdy1 strain were significantly higher than those of the other strains, and the vegetative growth was faster, demonstrating excellent biological characteristics.

### 3.3 Comparison of pathogenicity of different strains against WBPH

In order to compare the pathogenic effects of different *B. bassiana* on WBPH, we determined the pathogenicity of eight *B. bassiana* strains towards the insect. Experimental results revealed that there were significant differences (*p* < 0.05) in the pathogenicity of different *B. bassiana* strains against the third-instar nymphs of WBPH (Table 4). The cumulative corrected mortality of the WBPH was 54.06% ~ 77.67%, the muscardine cadaver rate was 31.11% ~ 57.78%, and the LT_50_ value was 5.12 ~ 8.07 days. After 10 days of treatment, there were three WBPH strains with a cumulative corrected mortality of over 70%, namely BEdy1, WJGP15, and JZ21004. Among them, the BEdy1 strain showed the highest cumulative corrected mortality against WBPH (*p* < 0.05), reaching 77.67% with an LT50 value of 5.12 days, demonstrating the highest pathogenicity. At the same time, after spraying with a high-concentration spore solution, muscardine cadavers appeared in all the strains. The muscardine cadaver rates of the BEdy1 and WJGP15 strains were 57.78 and 54.44%, respectively, which were significantly higher than those of the WJGP1 (44.44%), WJGP3 (38.89%), and CZ9 (26.67%) strains (*p* < 0.05). In summary, among the eight tested strains, the BEdy1 strain showed strong pathogenicity against the 3rd instar nymphs of the WBPH.

**Table 4:** Pathogenic effect to the 3rd-instar nymphs of *S. furcifera* treated with different strains.

Strains	Number of tested	Adjusted accumulative mortality rate (%)	Median lethal time LT_50_ (95% CI)	Rigid cadaver rate (%)
BEdy1	90	77.67 ± 1.56^a^	5.12(4.77 ~ 5.48)	57.78 ± 5.09^a^
WJGP1	90	65.89 ± 1.81^cd^	7.22(6.77 ~ 7.76)	44.44 ± 1.93^cd^
WJGP3	90	59.97 ± 2.55^e^	7.65(7.04 ~ 8.42)	38.89 ± 6.94^de^
WJGP5	90	69.42 ± 1.83^bc^	6.48(6.07 ~ 6.94)	52.22 ± 1.92^abc^
CZ9	90	54.06 ± 4.33^f^	8.07(7.42 ~ 8.94)	31.11 ± 8.39^e^
WJGP11	90	63.51 ± 2.50^de^	7.36(6.85 ~ 7.98)	45.55 ± 3.85^bcd^
WJGP15	90	70.57 ± 2.39^b^	5.88(5.49 ~ 6.31)	54.44 ± 5.09^ab^
JZ21004	90	71.76 ± 0.57^b^	6.21(5.82 ~ 6.65)	48.89 ± 5.09^abc^

### 3.4 Exploration of pathogenicity of *B. bassiana* BEdy1 strain against WBPH

**Figure 1:**
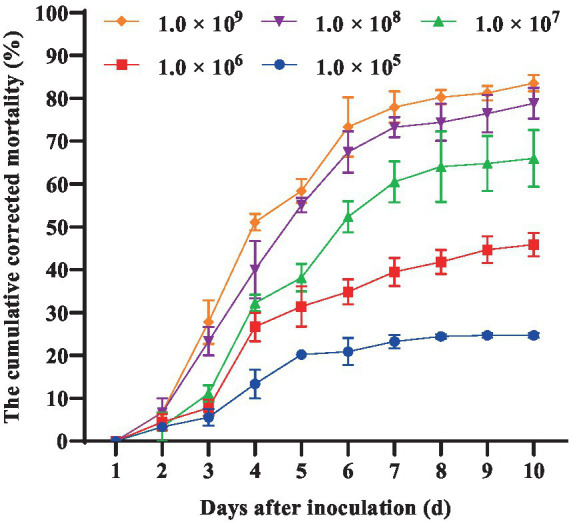
Pathogenicity of BEdy1 against the 3rd-instar nymphs of *S. furcifera.*

Furthermore, we conducted a detailed investigation into the pathogenicity of *B. bassiana* BEdy1 towards the WBPH. As shown in Figure 1, the cumulative corrected mortality of the 3rd instar nymphs of the WBPH was positively correlated with spore suspension concentration and treatment time of the *B. bassiana* BEdy1 strain. When treated with high concentrations of 1.0 × 10^9^ and 1.0 × 10^8^ spores/mL, a large number of WBPH died from the second day, and the cumulative mortality of the test insects after 10 days reached 83.54 and 77.67%, respectively; When treated with concentrations of 1.0 × 10^5^, 1.0 × 10^6^ and 1.0 × 10^7^ spores/mL, the mortality of the test insects increased significantly from the 3rd day, and the cumulative corrected mortality rates on the 10th day were 58.83, 45.85 and 28.20%, respectively. The pathogenicity of the *B. bassiana* BEdy1 strain to WBPH exhibits a time-dose relationship (Table 5), meaning that the higher the spore concentration of the treated insect, the smaller the LT_50_ value. Among them, the lethal time under the treatment of 1.0 × 10^9^ and 1.0 × 10^8^ spores/mL concentrations was 4.432 and 4.932 days, respectively. However, when the spore suspension concentration was between 1.0 × 10^5^ and 1.0 × 10^7^ spores/mL, the LT_50_ value of the test insect exceeded 6 days. Meanwhile, the LC_50_ value (10 days) of *B. bassiana* BEdy1 against the third-instar nymphs of WBPH was calculated to be 2.13 × 10^6^ spores/mL, with a 95% confidence interval of 8.81 × 10^5^ to 4.43 × 10^6^ spores/mL.

**Table 5:** LT_50_ of the BEdy1 strain against the 3rd-instar nymphs of *S. furcifera* under different spore concentrations.

Concentration (conidia/mL)	Adjusted accumulative mortality rate (10 d) (%)	Pathogenicity regression equation	LT_50_ (d)	95% confidence limit
1.0 × 10^9^	83.54	*Y* = 3.571X-2.309	4.432	4.139 ~ 4.721
1.0 × 10^8^	77.67	*Y* = 3.373X-2.338	4.932	4.610 ~ 5.260
1.0 × 10^7^	58.83	*Y* = 3.21X-2.578	6.356	5.943 ~ 6.822
1.0 × 10^6^	45.85	*Y* = 2.307X-2.271	9.648	8.568 ~ 11.293
1.0 × 10^5^	28.20	*Y* = 1.792X-2.306	19.360	14.655 ~ 30.932

### 3.5 Fluorescence microscopy observation of *B. bassiana* BEdy1 infecting WBPH nymphs

**Figure 2:**
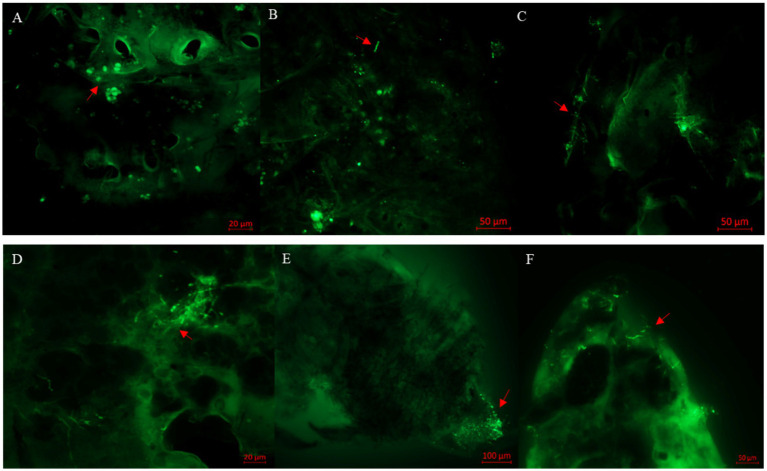
Observation of *S. furcifera* nymphs infected with *B. bassiana* BEdy1 under a fluorescence microscope. (A) Conidia on the cuticle of insects (12 h). (B) Germination of conidia (24 h). (C) Cross growth of hyphae after germination (36 h). (D) Propagation of some hyphae (60 h). (E) Penetration outside the post-abdomen by hyphae (84 h). (F) Head of the insect covered with hyphae (108 h); red arrow—conidia or hyphae.

After inoculation with BEdy1 strain for 12 h, a large number of viable spores aggregated near the abdominal sensors on the body surface (Figure 2A). After attaching to the body wall, the conidia were observed to start germinating into germ tubes at 24 h, with a small number germinating into hyphae (Figure 2B). At 36 h post-inoculation, the germinated hyphae extended horizontally on the insect’s surface, seeking suitable sites to penetrate the insect’s body wall (Figure 2C). By 60 h post-inoculation, some hyphae had proliferated within the insect body (Figure 2D). At 84 h post-inoculation, a large number of hyphae aggregated and grew at the abdominal end (Figure 2E), and a significant number of WBPH died at this stage. At 108 h post-inoculation, the hyphae had broken through the body wall and grown extensively on the insect’s head, including the compound eyes, vertex, and gena (Figure 2F).

### 3.6 Scanning electron microscopy observation of WBPH nymphs infected with *B. bassiana* BEdy1

**Figure 3:**
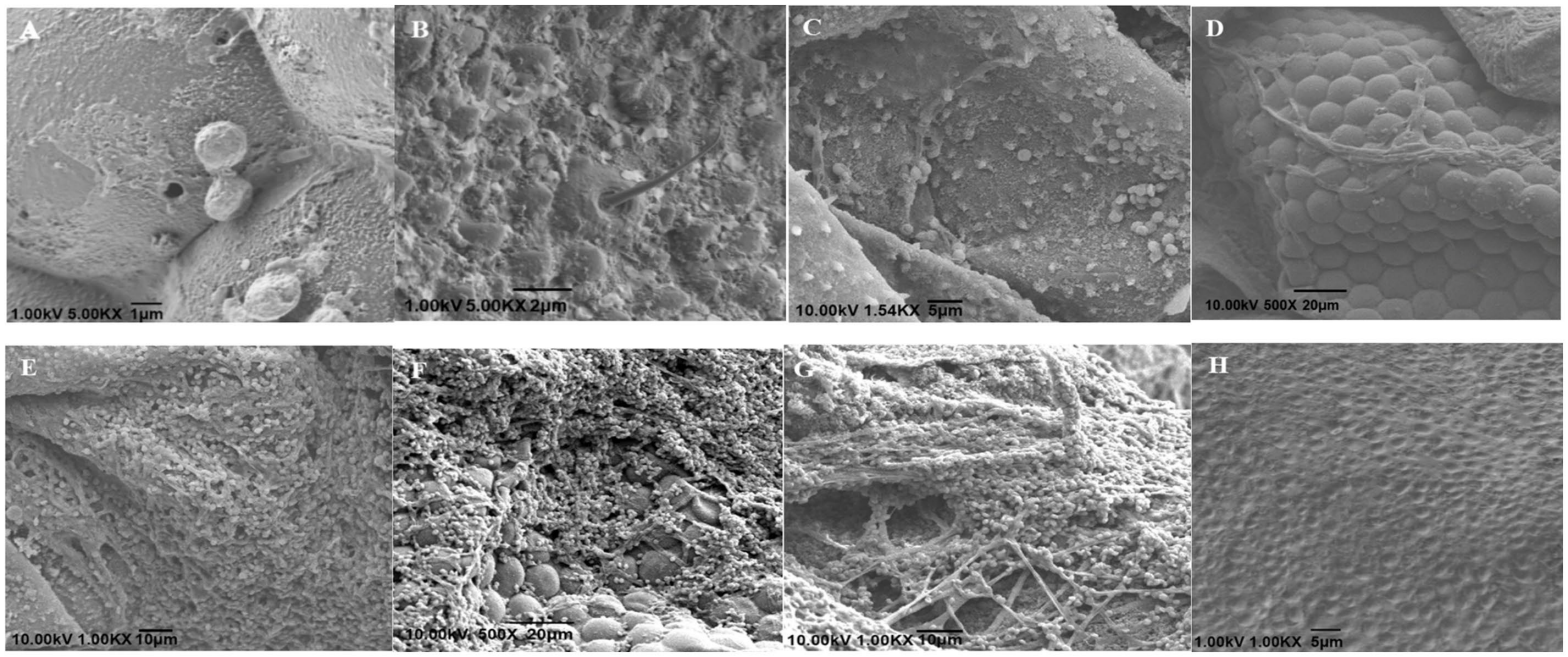
Scanning electron micrographs of *S. furcifera* nymphs infected with *B. bassiana* BEdy1. (A,B) Conidia on cuticle of the insect (12 h). (C) Germination and growth of conidia (24 h). (D) Some hyphae adhering to the compound eye (72 h). (E) Abdomen covered with hypha (96 h). (F) Hyphae on the compound eye (120 h). (G) Net-like structure among hypha (120 h). (H) The integument of the uninfected insect.

The scanning electron microscopy observation results are shown in Figure 3. Twelve hours after inoculation, the conidia were successfully attached to the small eyes and bristles near the head of the insect (Figures 3A,B). After 24 h of inoculation, a large number of conidia gathered in the abdomen of the insect and some spores had grown hyphae (Figure 3C). After 72 h after inoculation, the hyphae that penetrated the body wall were wound parallel to the compound eye (Figure 3D). After 96 h of inoculation, the mycelia grew and spread extensively in the abdomen of the insect (Figure 3E). After 120 h of inoculation, the head compound eye and bristles were extensively covered by the hyphae and conidia of *B. bassiana*, and the hyphae were intertwined to form a network structure (Figures 3F,G). At the same time, newly generated conidia were also observed to attach to the body wall, indicating that a suitable host is conducive to fungal survival and can promote the re-infection cycle of *B. bassiana*. In addition, the body wall of the uninfected WBPH was smoother (Figure 3H).

### 3.7 Histopathological observation of *B. bassiana* BEdy1 infection in nymphs of WBPH

**Figure 4:**
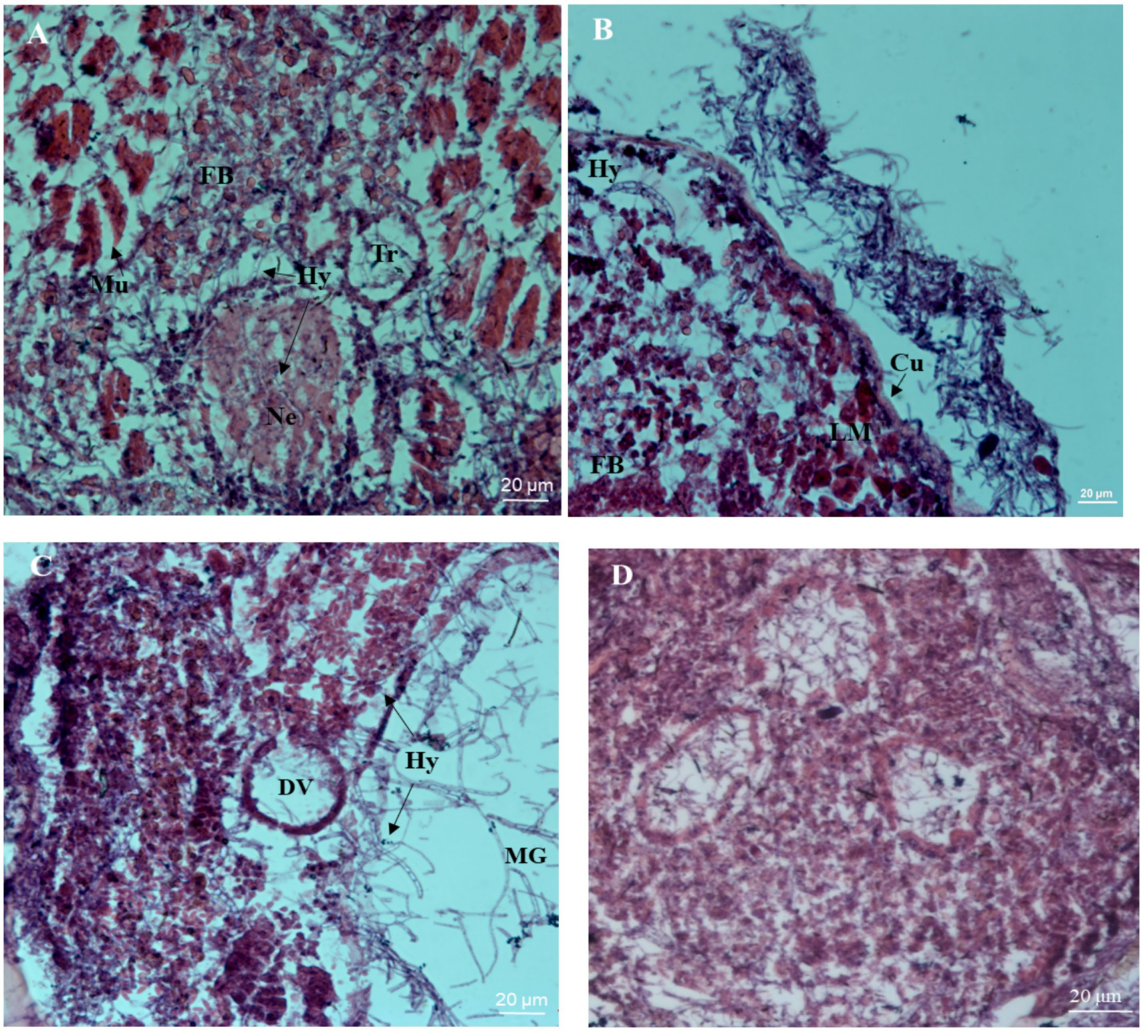
Histopathological micrographs of the nymph of *S. furcifera* infected with *B. bassiana* BEdy1. (A) Hypha (Hy) invaded the blood cavity, the atrophy of neuropile (Ne) and muscle (Mu) were split (60 h). (B) Cuticle (Cu) was separated from the inner tissue and hyphae appeared around the longitudinal muscle (LM) and fat body (FB) (72 h). (C) The dorsal vessel (DV), middle forg (MG), and visceral muscle were ruptured (96 h). (D) The inner structure of the uninfected insect was intact.

Tissue observation results at different time periods after inoculation are shown in Figure 4. After 60 h of inoculation, mycelial segments were observed in the internal tissues and organs of infected insects. The neuropile of the infected insect began to shrink, the trachea broke, and the surrounding muscle tissue appeared as loose strips (Figure 4A). After 72 h after inoculation, a large number of hyphae appeared in the hemocoel of the infected nymphs, with a loose arrangement of body wall muscles. The epidermis and internal structure of the nymphs were separated, and the hyphae grew outward and accumulated on the surface of the nymphs (Figure 4B), indicating that *B. bassiana* grew rapidly inside the nymphs. After 96 h of inoculation, many hyphae invaded the digestive tract and surrounded the dorsal blood vessels. The infected insect tissue became blurry and structurally incomplete, suggesting that it was the result of mechanical pressure generated by the hyphae or secretion of hydrolytic enzymes (Figure 4C). The tissue structure of the control insects was complete, and the muscle and other tissues were clearly visible (Figure 4D).

## 4 Discussion

The pathogenicity of *B. bassiana* against insects is a significant topic in the field of biological pest control. Currently, numerous studies primarily focus on screening new strains of *B. bassiana* for controlling the brown planthopper (*Nilaparvata lugens*), but there is minimal research on strains effective against the WBPH (Atta et al., 2020). Due to the heterogeneity of entomopathogenic fungi, different strains of *B. bassiana* exhibit varying levels of virulence against the same insect species (Arinanto et al., 2024; Basit et al., 2024). Therefore, screening for strains with high pathogenicity against the WBPH is a prerequisite for controlling this target pest.

Currently, bioassays are primarily utilized to screen for high-pathogenicity strains, with corrected mortality rates and median lethal time (LT_50_) serving as indicators to accurately assess the pathogenicity of different strains (Idrees et al., 2022). Studies have shown that a commercial *B. bassiana* preparation at a concentration of 4 × 10^9^ spores/mL can result in mortality rates of 79.8 and 71.6% for young and old larvae of the palm weevil, respectively (Erler and Ates, 2015). A commercial *B. bassiana* spore suspension at 1 × 10^8^ spores/mL can achieve a mortality rate of 96.67% for fourth-instar larvae of the pine sawyer beetle (*Monochamus alternatus*) on the 15th day (Guo et al., 2020). When treated with *B. bassiana* SG8702 at concentrations ranging from 1 × 10^6^ to 1 × 10^8^ spores/mL, the mortality rate of nymphs of the brown planthopper ranged from 32 to 91% (Feng and Pu, 2005). Another study indicated that 21 isolated strains of *B. bassiana* exhibited low pathogenicity against the brown planthopper, with a mortality rate of only 2–23%, although some strains reached a mortality rate of 62% after being passaged within the insect (Song and Feng, 2011). In this study, the LT_50_ value of the BEdy1 against the WBPH at a concentration of 1 × 10^8^ spores/mL was 5.12 days, and the corrected mortality rate reached 77.67% after 10 days, consistent with the results reported by Haider et al. (2021), indicating that the BEdy1 strain has high pathogenicity against the WBPH. Furthermore, there are significant differences in pathogenicity between the BEdy1 and other strains, which may be related to the biological characteristics of different strains (such as colony morphology, growth rate, and spore production) (Apirajkamol et al., 2023; Liu et al., 2008). Additionally, environmental conditions such as temperature, humidity, and the physiological stage of the host insect can significantly influence the infection process and outcomes. For example, high humidity and moderate temperatures have been found to enhance the virulence of *B. bassiana* against silkworms (Huang et al., 2014). Furthermore, the concentration and viability of spores, as well as the enzymatic activity of the fungus, play crucial roles in determining the success of infection. Therefore, further experiments will be conducted to investigate the enzymatic activity, spore viability, and the impact of environmental conditions on the pathogenicity of the BEdy1.

The process of fungal infection of insects involves several steps, including spore attachment to the host surface, germination and production of germ tubes, disruption of the host’s cuticle through mechanical pressure and extracellular enzymes, invasion into the host’s hemocoel, and colonization (Hong et al., 2024). In this study, we explored the infection process of BEdy1 on the WBPH using fluorescence microscopy and scanning electron microscopy (SEM). Fluorescence microscopy was primarily employed to observe the invasion of fungal cells into the insect body. As reported in the literature, after 1–5 d of infection with fluorescently labeled *B. bassiana*, there were few fungal cells in the peach aphid (*Myzus persicae*) body, but the number of hyphae increased after 6 d, and the hyphae invaded solid tissues after the insect’s death (Amnuaykanjanasin, 2013). In this study, the infection rate of BEdy1 on the WBPH was relatively rapid. Meanwhile, we used SEM to observe changes in the fungal infection on the insect’s body surface. As reported by Toledo et al. (2010), after 24–72 h of infection with *B. bassiana*, there were few conidia on the surface of the brown planthopper, but after 5–6 d, the entire host was invaded by hyphae. Additionally, Maha et al. (2021) found through SEM that after infection with *B. bassiana*, larvae of *Phlebotomus papatasi* showed significant hyphal growth on their abdomen and thorax after 96 h, and the infected adults had severely damaged bodies with noticeable hyphal growth on their heads (Maha et al., 2021). The results of our experiment revealed that BEdy1 began to invade after 24 h of inoculation, and hyphae penetrated the cuticle between 72 and 96 h, leading to massive insect mortality. These findings indicate that when *B. bassiana* BEdy1 infects the WBPH, the hyphae invade the insect body quickly and reproduce at a rapid rate, suggesting that BEdy1 can effectively control the WBPH in a timely manner.

The attachment of conidia is the first step in the parasitism of entomopathogenic fungi, and the smoothness of the insect’s body surface can affect the adhesion of fungal spores (Liu et al., 2010). Shah et al. (2003) suggested that the uneven and rough structural areas of the insect’s cuticle facilitate spore attachment. When Wang et al. (2006) infected the diamondback moth with *Metarhizium flavoviride*, they found that the conidia of *M. flavoviride* also attached to the folds on the insect’s body surface. Therefore, in this study, when BEdy1 initially infected insects, a large number of conidia aggregated near the abdominal sensilla and compound eyes of the insects. This may be because the surface of the WBPH’s abdomen, close to the sensilla, is relatively uneven, and the compound eyes are arranged in a honeycomb pattern, which is conducive to spore attachment.

When entomopathogenic fungi invade, they exhibit selectivity towards different regions of the insect’s cuticle, often growing parallel on unsuitable body surfaces (Wang and St Leger, 2005). In this experiment, we observed a phenomenon of horizontal parallel growth of *B. bassiana* hyphae, suggesting that *B. bassiana* BEdy1 selectively invades the WBPH and searches for suitable sites for infection during this process. Furthermore, research by Clarkson and Charnley (1996) indicated that the nutritional composition of the host’s cuticle determines the type of invasion structure formed by entomopathogenic fungi. Specifically, under conditions where the host’s cuticle nutrients support spore growth, spores germinate into germ tubes or hyphae, whereas under unfavorable conditions, spores germinate to form appressoria. Deng et al. (2008) found that when *B. bassiana* infects the Asian long-horned beetle (*Anoplophora glabripennis*), most spores penetrate the cuticle through germ tubes, while some invade through appressoria. Duan et al. (2017) reported that the *B. bassiana* NDBJJ-BFG penetrates the cuticle of the Colorado potato beetle through both germ tubes and appressoria after 48 h of infection. However, our study showed a different result: the conidia of BEdy1 invaded the nymph’s cuticle only through germ tubes and hyphae, and no appressoria were observed. This result is consistent with the infection mode of *Paecillomyces* sp. on the black scale insect (*Aleurocanthus spiniferus*) (Ma et al., 2000). This suggests that the nymph’s cuticle of the WBPH provides sufficient nutrients for *B. bassiana* BEdy1.

In this study, tissue sections of WBPH were prepared to observe the histopathological changes within the insect body after infection by BEdy1. Similar to the study by Ibrahim et al. (2019), where tissue sections of pear aphids were observed to reveal severe pathological changes in the hemolymph tissue caused by *B. bassiana*, Duan et al. (2017) found through sectioning that the Colorado potato beetle (*Leptinotarsa decemlineata*) experienced ruptured muscle tissue and deformed tracheae 72 h after infection by NDBJJ-BFG, leading to the insect’s death. By 96 h, the internal structure of the insect body was destroyed. In our current study, the tracheae of the infected insects were found to be fragmented, and the muscle tissue appeared blurred and structurally incomplete. These findings demonstrate that the tissue damage caused by BEdy1 to the WBPH is extremely severe, and they also reveal the pathogenic mechanism of BEdy1 from the perspective of the insect’s internal tissue structure.

## 5 Conclusion

In summary, this study screened a strain, BEdy1, from eight isolates of *B. bassiana*, which showed high pathogenicity against the WBPH. BEdy1 exhibited the highest cumulative mortality rate (77.67%) and the lowest LT50 (5.12 d) against the WBPH. Further investigation into the pathogenic process of BEdy1 revealed that it rapidly invades the insect’s cuticle in the form of germ tubes and hyphae, proliferates swiftly within the insect body, leading to tracheal rupture, blurred and structurally incomplete muscle tissue, and ultimately resulting in the insect’s death. BEdy1 can rapidly control the WBPH, providing a theoretical basis for achieving green pest management in rice.

## Data Availability

The datasets presented in this study can be found in online repositories. The names of the repository/repositories and accession number(s) can be found at: https://www.ncbi.nlm.nih.gov/, OM902633 https://www.ncbi.nlm.nih.gov/, OM902631 https://www.ncbi.nlm.nih.gov/, OM902632 https://www.ncbi.nlm.nih.gov/, OM902630 https://www.ncbi.nlm.nih.gov/, OM902629 https://www.ncbi.nlm.nih.gov/, OM902634.
